# Electrospun Environment Remediation Nanofibers Using Unspinnable Liquids as the Sheath Fluids: A Review

**DOI:** 10.3390/polym12010103

**Published:** 2020-01-04

**Authors:** Menglong Wang, Ke Wang, Yaoyao Yang, Yanan Liu, Deng-Guang Yu

**Affiliations:** School of Materials Science & Engineering, University of Shanghai for Science and Technology, Shanghai 200093, China; 172442574@st.usst.edu.cn (M.W.); yyyang@usst.edu.cn (Y.Y.); yananliu@usst.edu.cn (Y.L.)

**Keywords:** coaxial electrospinning, core-sheath nanofibers, environmental remediation, unspinnable liquid, nanocoating

## Abstract

Electrospinning, as a promising platform in multidisciplinary engineering over the past two decades, has overcome major challenges and has achieved remarkable breakthroughs in a wide variety of fields such as energy, environmental, and pharmaceutics. However, as a facile and cost-effective approach, its capability of creating nanofibers is still strongly limited by the numbers of treatable fluids. Most recently, more and more efforts have been spent on the treatments of liquids without electrospinnability using multifluid working processes. These unspinnable liquids, although have no electrospinnability themselves, can be converted into nanofibers when they are electrospun with an electrospinnable fluid. Among all sorts of multifluid electrospinning methods, coaxial electrospinning is the most fundamental one. In this review, the principle of modified coaxial electrospinning, in which unspinnable liquids are explored as the sheath working fluids, is introduced. Meanwhile, several typical examples are summarized, in which electrospun nanofibers aimed for the environment remediation were prepared using the modified coaxial electrospinning. Based on the exploration of unspinnable liquids, the present review opens a way for generating complex functional nanostructures from other kinds of multifluid electrospinning methods.

## 1. Introduction

Electrospinning, a simple and straightforward method for preparing nanofibers [1,2,3,4], is quickly moving forward along two important directions. One is the production on a large scale for potential commercial applications [5,6,7]. The other is the simultaneous treatment of multiple-fluid working fluids for generating nanofibers with complicated nanostructures [8,9,10,11]. 

**Figure 1:**
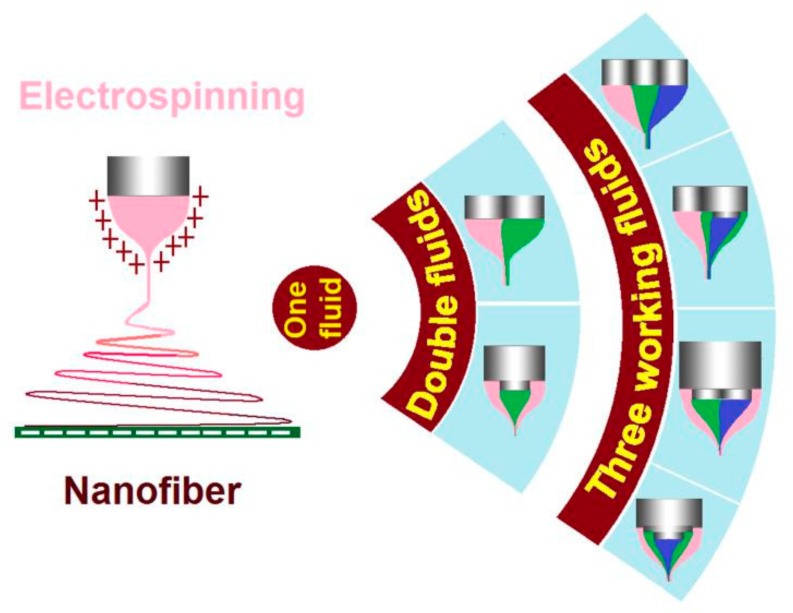
The development of electrospinning from the traditional one-fluid blending process to the double-fluid coaxial/side-by-side and three-fluid processes.

Shown in Figure 1, the traditional single-fluid blending electrospinning is differentiating into different sorts of double-fluid and three-fluid processes. Based on the spatial positions, there are two kind of double-fluid electrospinning, i.e., the coaxial electrospinning and the side-by-side electrospinning. As for the three-fluid processes, there are tri-axial process (an abbreviation of tri-layer working fluids organized in a coaxial manner), coaxial process with a side-by-side core, side-by-side process with one coaxial side, and tri-layer parallel side-by-side one. These advanced techniques should greatly expand the capability of electrospinning in generating a wide variety of complex nanostructures.

However, among all the complex nanostructures, the core-shell should be the most fundamental and the most important one [12]. It is not only because that many other complicated nanostructures can be viewed as a derivative of core-shell structure, but also this fundamental nanostructure has been broadly demonstrated to be extremely useful in designing and developing a wide variety of functional nanomaterials for applications in almost all the scientific fields [13,14]. Correspondingly, the methods for creating core-sheath structures are always highly desired in scientific fields. This should be one of the most important reasons that coaxial electrospinning [15,16], and also coaxial electrospraying [17,18,19,20,21], are receiving the increasing attention nearly from all the applied functional material fields.

## 2. The Modified Coaxial Electrospinning with an Unspinnable Sheath Fluid

In 2002, the first publication about core-shell structure was published based on electrospraying, or electrohydrodynamic atomization, a sister technique of electrospinning [22]. Later, in 2004, coaxial electrospinning was regarded as one of the most important three breakthroughs in this field [23]. In 2008, Moghe & Gupta gave the first review on coaxial electrospinning. Based on the previous publications before this time point, they concluded that the shell working fluids must be electrospinnable for successfully carrying out a coaxial process and for creating a core-shell nanostructure [24]. However, Yu, et al. broke this concept to develop a modified coaxial electrospinning, in which the sheath working fluid could be a pure solvent [12]. The modified process was successfully conducted to generate fibers from a concentrated polymer solution, which was impossible for a single-fluid process owing to the frequent clogging of the spinneret’s nozzle.

**Figure 2:**
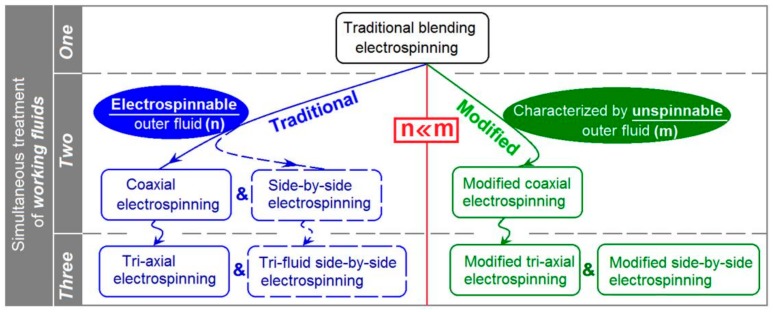
The differentiation of electrospinning from the traditional blending electrospinning to a series of double-fluid and tri-fluid electrospinning methods.

Shown in Figure 2, as the differentiation of traditional processes, it can be anticipated that the modified coaxial process can also differentiated into modified tri-axial process and even tri-layer side-by-side process. Most recently, modified tri-axial electrospinning with only one of the three working fluids having electrospinnability was demonstrated to be very useful in generating high quality core-shell nanofibers [25,26]. What is more, the functional sheath’s thickness can be easily manipulated through adjusting the fluid flow rate ratio in a suitable range.

**Figure 3:**
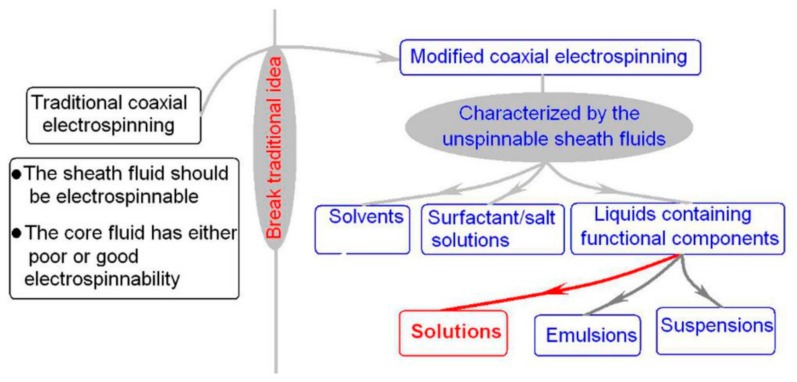
The differences between the traditional coaxial electrospinning and the modified coaxial electrospinning, and many kinds of unspinnable fluids can greatly enrich the electrospun nanoproducts.

Apparently, the involvement of unspinnable sheath fluid can greatly promote the capability of creating complex nanostructures for functional applications. This is because the spinnable filament-forming polymers are very limited, slightly over 100 kinds by estimation [27], but the un-spinnable liquids are numerous. Thus, the modified coaxial electrospinning provides those materials without filament-forming properties the previous opportunity to taking advantage of the large surface, high porosity and 3D web structure of electrospun nanofiber mats to exert their functional performances. Shown in Figure 3, those unspinnable liquids include solvents, surfactants or salt solutions, and liquids containing functional components. All the liquids are possible to be converted into electrospun nanofibers, regardless of they are solutions, emulsions, nano suspensions or even a slurry.

## 3. The Nanostructures Created by the Modified Coaxial Electrospinning and the Key Parameters for Carrying out the Working Processes

**Figure 4:**
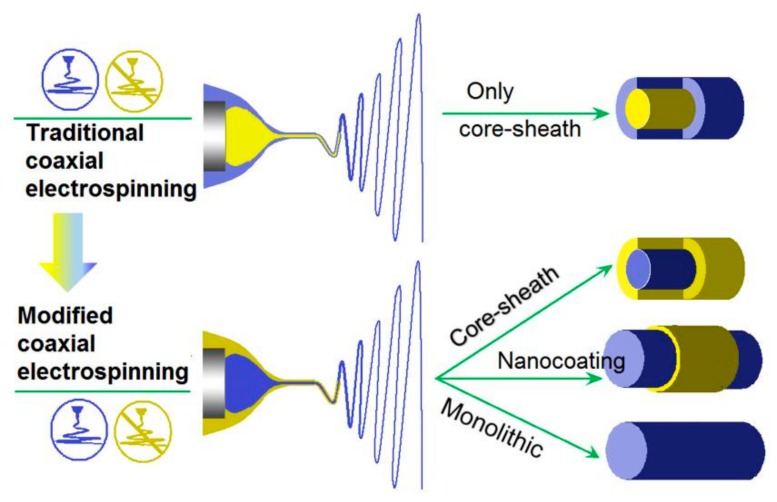
The nanostructures created by the traditional coaxial electrospinning and the modified one [28].

The advantages of modified coaxial electrospinning over the traditional one lies on not only the great treatment capability about unspinnable fluids prepared from numerous raw materials, but also in creating different kinds of nanostructures. Shown in Figure 4 is a comparison between the traditional coaxial and modified coaxial process in generating nanostructures [28]. The traditional coaxial electrospinning can only directly create solid core-shell structures, although which can be utilized to further prepare nanotubes and other derivatives [29]. In sharp contrast, the modified coaxial electrospinning can be exploited for producing core-shell structures, just as the traditional ones [30], but also can be utilized to prepare nanocoating on the core polymeric fibers [31]. What is more, when solvents are explored as the sheath working fluids, modified coaxial electrospinning can be utilized to stabilize the working process [32], keep the working processes from clogging, manipulate the nanofibers’ diameter without the additions of salt or other additives, and systematically improve the nanofibers’ quality.

In the traditional blending electrospinning, the working process is regulated by a series of variables, including the properties of the solution, experimental parameters and surrounding conditions [33,34,35,36,37,38,39]. The properties of solution further include the viscosity, conductivity, surface tension, molecular weight of polymer and dielectric constant; the operational variables include flow rate, electric field force, distance between needle and receiving screen, diameter and shape of needle, material composition and surface morphology of receiving screen, and so on; the surrounding parameters include temperature, humidity, wind speed, and maybe the vacuum state. But the most important thing is that these different parameters do not exert their impacts on the electrospinning process solely, they are not independent of each other, but interrelated. Thus, although electrospinning is a simple one-step processes, but it is very difficult to be optimized for robust and repetitive preparation.

**Figure 5:**
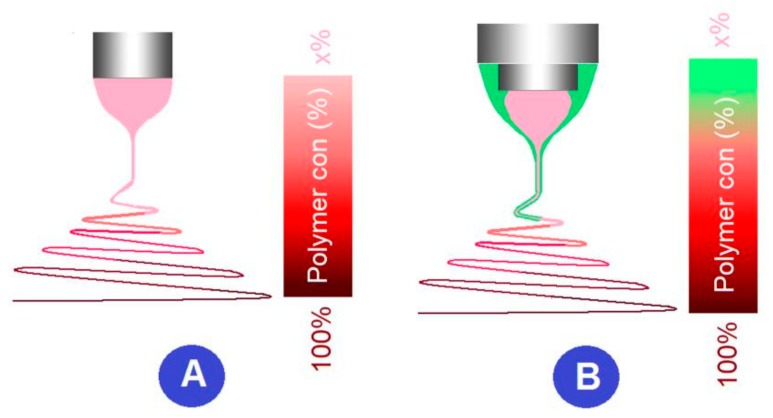
A diagram showing the solidification processes during (**A**) the traditional and (**B**) modified coaxial electrospinning using a solvent as a sheath working fluid [40].

No matter how many fluids are simultaneously electrospun using the multiple-fluid electrospinning processes, the above-mentioned parameters will similarly influence the working processes and the final products’ quality. The additional key parameter for the modified coaxial electrospinning should be the flow rates of sheath/core working fluids or the sheath-to-core fluid flow rate ratio. This is particularly vital for the modified coaxial electrospinning, in which solvent is exploited as a sheath fluid. Shown in Figure 5 is a diagram about the comparison between a single-fluid electrospinning and a modified process with a sheath solvent [40]. Compared with the former, the latter has a layer of solvent around the core spinnable polymeric solution during the previous stage of the whole electrospinning process, i.e., the Taylor cone, the straight fluid jet, and partial the unstable region. On one hand, the solvent can lubricate the spinneret to prevent clogging, can decrease the negative drawing forces from the capillary’s surface and the capillary forces, and can keep the core fluid from disturbance by the environmental changes. On the other hand, the sheath solvent may result in a failure preparation of linear nanofibers or even a failure of solid products [41].

The sheath-to-core fluid flow rate ratio *R* can be expressed as *R = F_s_/F_c_*, where *Fs* and *F_c_* are flow rates of sheath and core working fluids, respectively. When *R* is manipulated in a suitable range, the sheath solvent will make positive influences on the whole coaxial working process and benefit the quality of final solid nanofibers. However, when *R* value is beyond the limitation, i.e., the sheath solvent flow rate is too large, the beads-on-a-string or spindles-on-a-string may be generated. This is because the sheath solvent can’t be completely exhausted in time and in turn influence the solidification of the core fluid [42].

## 4. The Nanofibers Prepared from Unspinnable Liquids for Environment Remediation

Based on coaxial electrospinning, the applications of electrospun nanofibers from unspinnable fluids for pharmaceutical applications have been broadly reported, which can provide different kinds of drug-controlled release profiles such as immediate release, sustained release and multiple-phase release [43,44,45,46]. However, the publications about their environmental applications are still very limited, the published jobs have demonstrated the usefulness of this advanced electrospinning method in creating novel functional nanostructures for removing heavy metal ions from the polluted water and for antibacterial applications to keep a healthy environment.

### 4.1. Zein Nanoribbons from Sheath Surfactant Solution Electrospinning for Environmental Remediation

**Figure 6:**
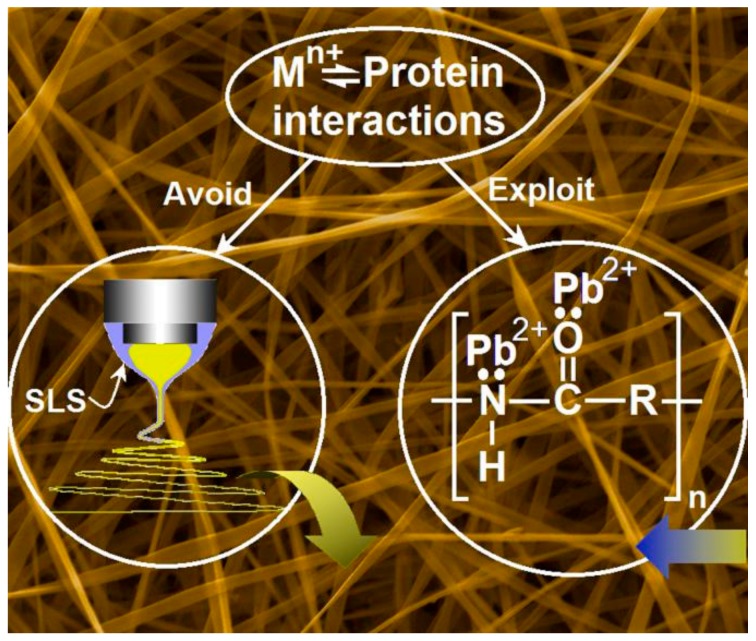
The mechanisms for the preparation of electrospun zein nanoribbons using unspinnable surfactant solution as a sheath fluid, and their applications in removing the lead ions from polluted water. Reprinted with permission from [49].

It is well-known that biomacromolecules have the potential to become fine adsorbents for the removal of heavy metals [47,48]. Particularly, protein molecules contain numerous groups with lone pairs of electrons and partial negative charges, and these groups allow protein molecules to absorb metal ions through electrostatic interaction and/or chelation. Zein is a typical plant protein, which is naturally abundant and eco-friendly. Shown in Figure 6, the interactions between the metal ions and the protein molecules comprise both the mechanism for modified coaxial electrospinning with unspinnable sheath fluid and mechanism for the ion removement from polluted water. On one hand, during the modified coaxial process, the sheath solution can eliminate the Mn^+^-protein interactions for avoiding the frequent clogging, and thus ensure a continuous and robust preparation process. On the other hand, during the applications, this interaction is exploited to absorb lead ions from the polluted water for environmental remediation.

**Figure 7:**
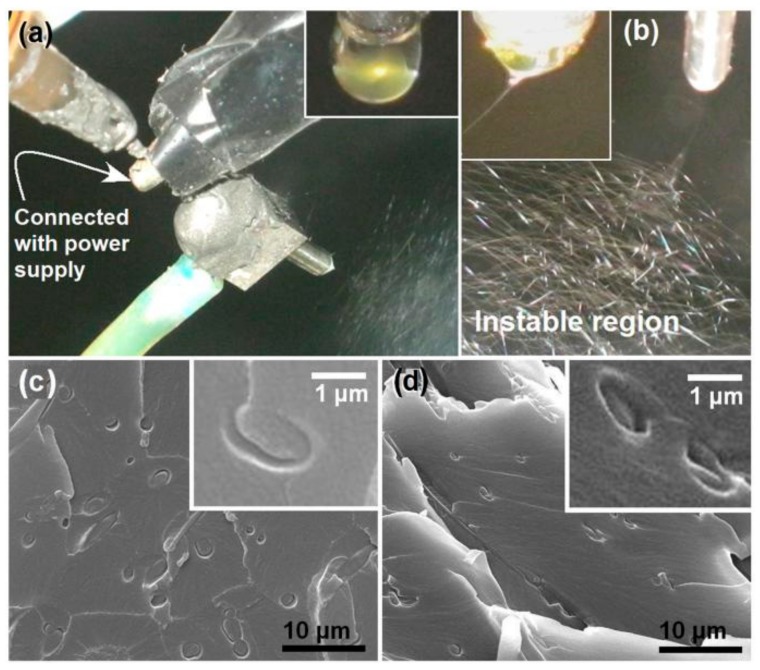
The working processes of modified coaxial electrospinning (**a**,**b**) using unspinnable surfactant solution as a sheath working fluid and the prepared zein nanoribbons (**c**,**d**). Reprinted with permission from [49].

The difference between the traditional single-fluid blending process and the multiple-fluid process mainly lie in how to guide the fluids in an organized manner. Shown in Figure 7a,b are digital photos about the modified coaxial methods for creating zein nanoribbons. The upper-right inset shows a core-shell droplet, in which the core spinnable zein solution consisting of 30 g zein in 100 mL of a 75%/25% (*v/v*) ethanol/water mixture was surrounded by a transparent and unspinnable sheath fluid composed of 0.5% (*w/v*) sodium lauryl sulfate in 75%/25% (*v/v*) ethanol/water. An enlarged image shown in Figure 7b clearly tells the typical three steps of electrospinning, i.e., the compound Taylor cone (the upper-left inset), the straight fluid jet, and the bending and whipping regions with enlarged loops in the instable region.

Cross-sections of different zein nanoribbons were prepared, which are compared in Figure 7c,d. The former image is zein ribbons prepared using the single-fluid electrospinning. The latter image is zein ribbons created using a modified coaxial electrospinning, in which unspinnable SDS ethanol aqueous solution was exploited as a sheath working fluid. By estimation, the surface area of zein ribbons from the coaxial process was enlarged 2.29 times than those from the single-fluid process, which was favorite for lead ion adsorption. By exploiting the favorable interactions between metal and protein, the zein nanoribbon mats were used to treat Pb^2+^-polluted water. Adsorption results indicated that the adsorption process can be described using the pseudo-second-order model. Isotherm data fitted well to the Langmuir isotherm model, with a maximum adsorption of 89.37 mg/g for the nanoribbons prepared from the modified coaxial process. Desorption results showed that the adsorption capacity can remain up to 82.3% even after 5 cycles of re-use. These positive results apparently demonstrated that the unspinnable working fluids utilized in the modified coaxial electrospinning were able to stabilize the preparation process, to further downsize the nanoproducts’ width, and to improve their functional performances in environmental remediation.

### 4.2. Pure Solvent as a Sheath Fluid to Create Oligomer-Loaded Functional Nanofibers for Removing Mercury Ions

For the traditional single-fluid electrospinning, the filament-forming polymeric matrices must have enough molecular weight to support enough physical entanglements for forming electrospun fibers within their electrospinnable windows [50]. Few reports can be found on the directly electrospun nanofibers of oligomers because they haven’t enough molecular weight and in turn no enough physical entanglements in their working fluids. These oligomers solutions are often un-electrospinnable and can’t be converted into nanofibers. 

However, in a recent investigation, an oligomer Poly(2-aminothiazole) (PAT) was synthesized, and then this functional oligomer solution (0.1 g of PAT was dissolved in a mixed solvent of 2 mL DMAC and 2 mL ethanol) was mixed with a cellulose acetate (CA, 1.44 g of CA was dissolved in 8 mL of acetone) solution to create composite nanofibers. The sheath fluid was pure solvent acetone, which was pumped quantitatively through another booster to another inlet of a concentric spinneret for a smooth working process.

**Figure 8:**
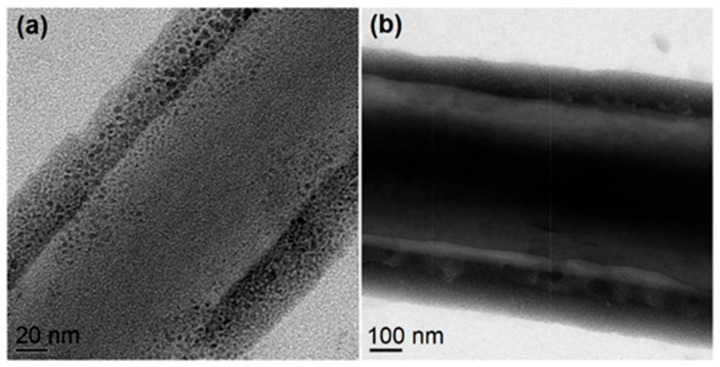
Both (**a**) and (**b**) are representative TEM images of 0.1 g PAT/1.44 g CA nanofibers with unexpected core-shell nanostructures. Reprinted with permission from [50].

Figure 8 shows two representative high-magnification TEM images of the PAT/CA fibers, which clearly present their core-sheath nanostructures. The sheath thickness ranged from 35 to 205 nm, and the core thickness ranged from 105 to 480 nm. The sheath section was mainly composed of small PAT nanoparticles with only few nanoparticles appeared in the core section. This phenomenon is beyond anticipation because there was no any solutes in the sheath working fluid. The resultant core-shell structures should be a reason of solid phase separation during the modified coaxial processes. The concrete forming mechanism is still waited to be further disclosed.

The Hg(II) adsorption properties of the PAT/CA composite nanofiber mat from polluted water was tested. The adsorption capability was highly dependent on the pH value of solution, with a maximum at pH 6.5. The isotherm data fitted well to the Langmuir isothermal model. The adsorption kinetics data fitted well to the pseudo-second-order model. At a temperature of 298 K, the maximum adsorption capacity of 177 mg/g for PAT/CA fiber membrane with a very low PAT percentage (6.5 wt.%) was calculated. Through conversion, the adsorption capacity of the PAT in the composite fiber membrane is much larger than that of the nanoparticle-type PAT. The desorption experiments revealed that the desorption percentage remained at 80.2% after being used for three times. Here the unspinnable sheath solvent smoothed the electrospinning processes to prevent the spinneret clogging of CA, resulted in solid phase separation to lead to a surface distribution of functional ingredient, i.e., PAT particles, and thus provided an improved functional performance of removing Hg(II) from water. Certainly, the PAT-loaded nanofibers, as a non-woven mat, were more convenient than the PAT powders themselves for environmental remediations and less possibility of secondary pollutions due to nanoparticles themselves.

### 4.3. Nanocoating of Inorganic Materials on Polymeric Fibers for Antibacterial Applications Using Unspinnable Salt Solution as a Sheath Fluid

Through the modified coaxial electrospinning, it is possible to directly distribute the inorganic particles on the surface of polymeric nanofibers, in which nanosuspension can be explored as the sheath working fluid. Unfortunately, the inorganic nanoparticles are very easy to precipitate during the working processes, which may stop the continuous preparation. However, a combined strategy based on the modified coaxial electrospinning using salt solution as a sheath working fluid can overcome this issue. 

**Figure 9:**
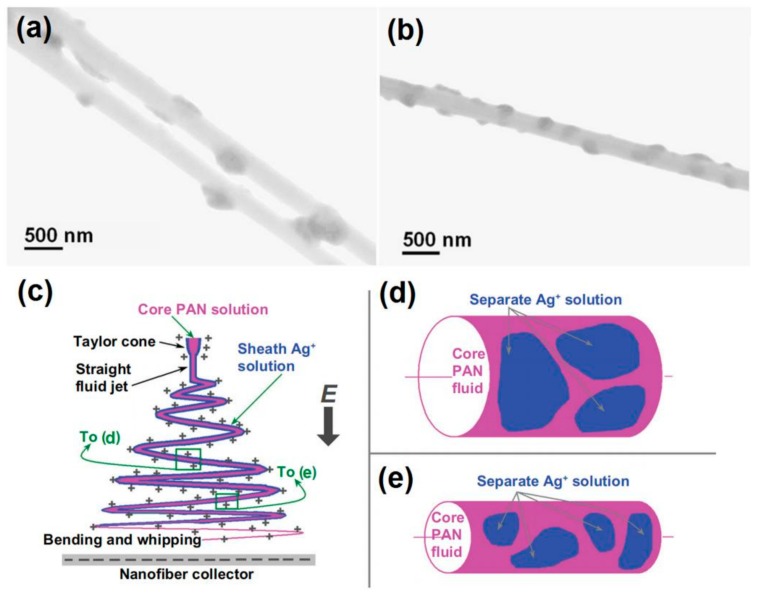
The Ag nanoparticles coating nanofibers (**a**,**b**) and the mechanisms for fabricating inorganic nanoparticles distributed PAN hybrids for antibacterial applications (**c**–**e**). Reprinted with permission from [51].

With a solution of 15% (*w/v*) polyacrylonitrile (PAN) in N, N-dimethylacetamide (DMAc) as the electrospinnable core working fluid, a 10% (*w/v*) AgNO_3_ DMAc solution was employed as a sheath fluid, a modified coaxial process was successfully carried out to create the salt coating PAN nanofibers. The electrospun nanofibers were then exposed to 254 nm ultraviolet light for 24 h to reduce the Ag^+^ to Ag nanoparticles (NPs). The prepared products are shown in Figure 9a,b. Both nanofibers have kind linear morphology with the Ag NPs uniformly distributed on the PAN surface. This is very useful for the Ag NPs to exert their functional performances.

The influence of the key parameter, i.e., the sheath-to-core fluid flow rate ratio was investigated. The results demonstrated that the smaller flow rate of AgNO_3_ solution of 0.1 was not able to follow the core PAN fluid jet during the fast bending and whipping process. This results in a separation of the sheath AgNO_3_ solution and a non-uniform deposition of AgNO_3_ on the surface of nanofibers (Figure 9c,d). Thus, a higher sheath-to-core fluid rate ratio of 0.2 was selected to carry out the experiments, which resulted in a more homogeneous distribution of AgNO_3_ on the surface of nanofibers. When the Ag^+^ on the surface of PAN nanofibers was reduced to Ag NPs, the nanofibers from a larger sheath fluid flow rate ratio had a more uniform but smaller size, and a tighter distribution than those from a smaller ratio (Figure 9c,e). It is anticipated that reduction of Ag^+^ to Ag NP on the surface of nanofibers should be beneficial to long-term stability of the system and prevent Ag leaching from the fibers. These Ag NPs-loaded nanofibers showed fine antibacterial performances and should be candidates for friendly ecological environmental applications.

The popularity of electrospun nanofibers has a close relationship with their small diameters, huge surface area and large porosity [52,53,54]. However, for a single-fluid electrospinning process, the treated fluids must be electrospinnable to keep the formation of solid linear nanofibers, or they will be degraded into particles or even wet films [40,55]. The modified coaxial electrospinning, with an unspinnable sheath fluid on a spinnable core fluid, can’t be utilized to prepare some advanced nanostructures presently such as hollow fibers from a traditional coaxial processes [56,57], which can be viewed as a special core-shell structure with an empty core. However, all the liquids, containing or not the filament-forming polymers, can be converted as a thin film on the solid core fibers to take advantages of the nanofibers’ unique properties. This give a hint on many new possibilities in future. There are many publications about the environmental applications of electrospun composite nanofibers, in which the guest functional ingredients scattered within host polymer matrices [58,59,60,61]. With the valuable hints from these investigations, new types of nanofibers can be fabricated using the similar raw materials for an improved functional performance. The functional ingredients can be prepared as their owns’ working fluids in the sheath section to superficialize them on the solid hybrid nanofibers. Meanwhile, the pure polymers core may increase the mechanical properties of the core-sheath structures. 

## 5. Conclusions and Perspectives

As the fast development of electrospinning, modified coaxial electrospinning is upgraded from the traditional one and shows strong vitality. Its usefulness lies in both stabilizing working process for creating high quality monolithic nanofibers, but also in generating solid core-shell nanostructure and nanocoating. What is more, almost any kinds of functional raw materials, regardless of filament-forming property, can be treated into nanofibers to exert their functional performances by taking advantages of the unique properties of electrospun non-woven mats. The above-mentioned several examples demonstrated that the modified coaxial processes can be utilized to manipulate the nano products’ shape and size facilely, to incorporate oligomer into the nanofibers, and to coat inorganic salt on the surface of polymeric nanofibers. These preparations are impossible using the traditional processes. Based on the combination of modified coaxial electrospinning and the tremendous active ingredients for treating pollutants in our environment, a new platform can be built to develop more and more functional nanostructures for environmental remediation. 

The new nanomaterials and the corresponding applications resulted from the modified coaxial electrospinning using unspinnable liquid as a sheath working fluid is still at its infant time period. It is anticipated there are more and more possibilities in future, an unspinnable fluid can be treated with one or more spinnable fluids in many ways such as as tri-axial [62,63] and side-by-side [64]. Electrospinning and the electrospun nanofibers may find their applications in almost all the environmental issues such as gas pollution, water pollution and solid waste. They can also find applications for a better ecological environment, such as producing antibacterial materials, anti-UV materials, noise reduction and electromagnetic shielding [65]. Similarly, modified coaxial electrospinning and the corresponding nanofibers and nanostructures can upgrade these applications and generate new materials from an even wide selections of raw materials such as inorganic NPs, carbon nanotube, graphene and small molecules. In traditional monolithic nanofibers, the active ingredients are homogeneously distributed all over the polymeric matrices, means that most of them are buried into the nanofibers and can’t play their roles for removing the pollutants [66]. With the unspinnable solution as a sheath working fluid, a wide variety of functional ingredients (such as cyclodextrin) can be effectively distributed on the surface of polymeric nanofibers [67,68], and thus their functional performances on environmental remediation can be easily enhanced. Certainly, the modified coaxial electrospinning can further expand its capability of creating novel functional material through a combination with other spinning methods or numerous traditional physical and chemical manners [69,70].

Just as the traditional electrospinning, one of the most important challenges for the modified coaxial electrospinning is to create nanofibers on a large scale [71,72,73]. Another important challenge is get more and more knowledge about the special working processes [74], here the key point is about the unspinnable sheath fluid’s role during the transmission of solvents through air-fluid-fluid interfaces and the related solidification mechanism of electrospun nanostructures. Certainly, the structure-performance relationships for environmental and also other applications based on the nanoproducts prepared using modified coaxial electrospinning should be a very interesting topic deservin.
